# Quantitative analysis of *N*‐acylphosphatidylethanolamine molecular species in rat brain using solid‐phase extraction combined with reversed‐phase chromatography and tandem mass spectrometry

**DOI:** 10.1002/jssc.201600172

**Published:** 2016-06-07

**Authors:** Alexander Triebl, Sabrina Weissengruber, Martin Trötzmüller, Ernst Lankmayr, Harald Köfeler

**Affiliations:** ^1^ Core Facility for Mass Spectrometry, Center for Medical ResearchMedical University of GrazGrazAustria; ^2^ Institute of Analytical Chemistry and Food ChemistryGraz University of TechnologyGrazAustria

**Keywords:** Mass spectrometry, *N*‐acylphosphatidylethanolamine, Solid‐phase extraction

## Abstract

A novel method for the sensitive and selective identification and quantification of *N*‐acylphosphatidylethanolamine molecular species was developed. Samples were prepared using a combination of liquid–liquid and solid‐phase extraction, and intact *N*‐acylphosphatidylethanolamine species were determined by reversed‐phase high‐performance liquid chromatography coupled to positive electrospray tandem mass spectrometry. As a result of their biological functions as precursors for *N*‐acylethanolamines and as signaling molecules, tissue concentrations of *N*‐acylphosphatidylethanolamines are very low, and their analysis is additionally hindered by the vast excess of other sample components. Our sample preparation methods are able to selectively separate the analytes of interest from any expected biological interferences. Finally, the highest selectivity is achieved by coupling chromatographic separation and two *N*‐acyl chain specific selected reaction monitoring scans per analyte, enabling identification of both the *N*‐acyl chain and the phosphatidylethanolamine moiety. The validated method is suitable for the reliable quantification of *N*‐acylphosphatidylethanolamine species from rat brain with a lower limit of quantification of 10 pmol/g and a linear range up to 2300 pmol/g. In total, 41 *N*‐acylphosphatidylethanolamine molecular species with six different *N*‐acyl chains, amounting to a total concentration of 3 nmol/g, were quantified.

**Abbreviations**
NAE: *N*‐acylethanolamine
NAPE: *N*‐acylphosphatidylethanolamine
PC: glycerophosphocholine
PE: glycerophosphoethanolamine
SRM: selected reaction monitoring

## 1 Introduction

*N*‐Acylphosphatidylethanolamines (NAPEs) are triacylated phospholipids derived from phosphatidylethanolamine (PE) with a third, *N*‐linked fatty acyl chain 1. NAPE synthesis is catalyzed by *N*‐acyltransferase that transfers a fatty acyl chain from the *sn‐*1 position of phosphatidylcholine (PC) to the amino group of a PE molecule. The primary pathway of NAPE degradation is generation of *N*‐acylethanolamine (NAE) by a Ca^2+^‐dependent enzyme with phospholipase D activity (NAPE‐PLD). Other pathways are by a NAPE‐selective hydrolase with phospholipase 1/2 activity, or degradation to phospho‐NAE and diacylglycerol by an enzyme with phospholipase C activity 2, 3. NAEs are important signaling molecules, the most well‐known of which is anandamide (*N*‐arachidonoylethanolamine), an agonist of cannabinoid receptors. The different NAEs have a variety of biological effects, among them control of food intake, inflammation, pain sensation, and neuroprotection 4. NAPEs not only serve as precursors to NAEs, but also have biological functions of their own. Increasing NAPE levels together with their product NAEs after neuronal damage 5, 6, 7 are indicative of their involvement in a neuroprotective mechanism 8, 9. Postprandial increase of NAPE levels and inhibition of food intake by their administration, may suggest a potential anorectic role 10, 11, although this is subject of controversy and has not yet been clearly shown 1.

Tissue levels of NAPE in mammals are in the range of a few nmol/g 1, and NAPEs are usually not covered by “comprehensive” HPLC, SFC, or shotgun mass spectrometric lipidomic approaches 12, 13, 14, 15, 16, 17, 18, 19, 20, but rather require specialized approaches in terms of sample preparation and identification. By hydrolysis of the *sn*‐1 and *sn*‐2 bonds of NAPE the resulting glycerophospho‐NAE species can be determined either by LC–MS 9, 21, GC–MS 22, 23 or HPLC coupled to fluorescence detection 24. However, this approach groups all species with the same *N*‐acyl chain together without the possibility for individual detection of intact NAPE molecular species. Full scan methods 6, 7, 25 or single ion monitoring methods 26 can only deliver elemental compositions, with no identification of substructures such as the *N*‐acyl chain. Selected reaction monitoring (SRM) in negative ion mode, with the carboxylate anion 27, 28 or neutral loss 29, 30 of the *sn*‐2 fatty acyl chain as the product ion, is only able to identify the *sn*‐2 fatty acyl, and further targeted tandem MS experiments are necessary to determine the *N*‐acyl chain 31. Positive ion mode, either with precursor scanning 32 or neutral loss scanning specific for the *N*‐acyl chain and combined with FIA, has also been employed for NAPE detection 33, 34.

Here, we present a novel approach for selective quantification of NAPE compounds at the level of individual species including *N*‐acyl chain determination, using LLE and SPE for sample purification, and detection by a combination of RP‐HPLC and triple quadrupole MS. The highest selectivity is ensured by coupling chromatographic separation and two *N*‐acyl chain specific SRM scans per analyte, enabling identification of both the *N*‐acyl chain and the phosphatidylethanolamine moiety.

## 2 Materials and methods

### 2.1 Chemicals and lipid standards

Methanol, acetonitrile, methyl‐*tert*‐butyl ether (MTBE), dichloromethane, palmitoyl chloride, heptadecanoyl chloride, stearoyl chloride, oleoyl chloride, 4‐dimethylaminopyridine, ammonium hydrogen carbonate, ammonium formate, 2‐propanol, formic acid, and acetic acid were purchased from Sigma–Aldrich (St Louis, MO, USA), and pyridine was purchased from VWR (Radnor, PA, USA). Deionized water was obtained from a MilliQ Gradient A10 system (Millipore, Billerica, MA, USA).

1,2‐Di‐(9*Z*‐octadecenoyl)‐*sn*‐glycero‐3‐phosphoethanolamine‐*N*‐nonadecanoyl (PE 18:1/18:1‐N‐19:0) and 1,2‐dioleoyl‐*sn*‐glycero‐3‐phosphoethanolamine‐*N*‐arachidonoyl (ammonium salt) (PE 18:1/18:1‐N‐20:4) were purchased from Avanti Polar Lipids (Alabaster, AL, USA) and l‐α‐phosphatidylethanolamine from Larodan Fine Chemicals AB (Malmö, Sweden). Lipid standard stock solutions at concentrations of 1 mmol/L were prepared in chloroform/methanol (1:1, v/v).

### 2.2 Synthesis of *N*‐acylphosphatidylethanolamine standards

NAPE standards were synthesized as described by Guo et al. 21, modified after Höfle et al. 35. 1 mL dichloromethane was mixed with 0.25 μmol l‐α‐phosphatidylethanolamine and 25 μL pyridine diluted 1:25 with dichloromethane, and the mixture was vortexed for 10 s. 6.25 μmol of 4‐dimethylaminopyridine and 6.5 μmol of the respective acyl chloride (palmitoyl, heptadecanoyl, stearoyl or oleoyl chloride) were added and the reaction was allowed to take place for 18 h at 21°C. The mixture was transferred to a 12 mL glass tube and quenched with 1 mL saturated aqueous NH_4_HCO_3_ solution. Phases were separated by centrifugation (3 min, 1350 g, 21°C) and the upper aqueous phase was discarded. This process was repeated and the lower NAPE containing phase was collected and purified by LLE and SPE as described below. 1 nmol of commercially available NAPE standards PE 18:1/18:1‐N‐19:0 and PE 18:1/18:1‐N‐20:4 were spiked to 100 μL of the synthesized heptadecanoyl NAPEs and their concentrations were calculated from the mass spectrometric peak areas.

### 2.3 LLE

LLE was performed according to Matyash et al. 36 with some modifications. 100 mg rat brain was spiked with 50 pmol PE 18:1/18:1‐N‐19:0 internal standard in a glass tube with a Teflon‐lined cap. 1.5 mL methanol and 5 mL MTBE were added and the mixture was homogenized using a rotor‐stator homogenizer for 10 s at 10 000 rpm. After 10 min overhead shaking, phase separation was induced by addition of 1.25 mL deionized water. The mixture was shaken for another 10 min and centrifuged (3 min, 1350 *g*, 21°C). The upper organic phase was transferred to a new tube and the lower phase was re‐extracted with 2 mL of MTBE/methanol/water (10:3:2.5, v/v/v). The combined upper phases were evaporated in a vacuum centrifuge and resuspended in 1 mL of MTBE/chloroform/acetic acid (98:2:0.2, v/v/v) for further SPE purification.

### 2.4 SPE

SPE was performed on Strata SI‐1 Silica (55 μm, 70 Å), 500 mg/3 mL tubes (Phenomenex, Aschaffenburg, Germany) in a Varian VacElut 20 Manifold (Agilent, Waldbronn, Germany) as described 9 with minor modifications (Table 1). A constant negative pressure of 800–820 mbar was maintained to ensure reproducible results and increase sample throughput. The NAPE containing fraction was collected, evaporated in a vacuum centrifuge and reconstituted in 200 μL of 2‐propanol/chloroform/methanol (90:5:5, v/v/v).

**Table 1:** Solid phase extraction method

Step	Solvent	Volume
	composition (v/v/v)	(mL)
Conditioning	MTBE/chloroform/acetic acid (98/2/0.2)	7.5
Sample loading	MTBE/chloroform/acetic acid (98/2/0.2)	1.0
Wash I	MTBE/chloroform/acetic acid (98/2/0.2)	7.5
Wash II	MTBE	5.0
NAPE elution	MTBE/chloroform/methanol (50/20/30)	9.0

### 2.5 Chromatography

NAPE species were separated on a Waters Acquity UPLC BEH C_8_ (1.7 μm, 1 mm x 100 mm; Milford, MA, USA) column, thermostatted to 50°C in an Advance UHPLC system (Bruker Daltonics, Bremen, Germany). Mobile phase A was deionized water containing 1 vol% of 1 M aqueous ammonium formate and 0.1 vol% formic acid; mobile phase B was acetonitrile/2‐propanol (5:2, v/v) with the same concentrations of additives. The binary gradient started with 85% B and linearly increased to 100% B over 15 min. 100% B were held for 3 min before the column was re‐equilibrated with 85% B for another 12 min. The flow rate was 100 μL/min, the autosampler tray was kept at 10°C and 2 μL of sample were injected.

### 2.6 MS

NAPEs were analyzed on an Evoq Elite ER (Bruker Daltonics, Bremen, Germany) triple quadrupole mass spectrometer in positive polarity, controlled by MSWS version 8.2. A total of 79 NAPE molecular species were analyzed simultaneously using two SRM transitions per analyte (see Section 3.4 and Supporting Information Table S1 for details). Retention time windows were set to 3 min centered at the expected retention time, resulting in a median dynamic scan time of 25 ms per transition. Q1 and Q3 mass windows were 2 *m/z*, and argon with a pressure of 1.5 mtorr was used as collision gas. Source parameters were as follows: Spray voltage, 4800 V; cone temperature, 300°C; cone gas flow, 20; heated probe temperature, 125°C; heated probe gas flow, 15; nebulizer gas flow, 20; exhaust gas, on.

### 2.7 Data analysis

MS Data Review v. 8.2 and Microsoft Excel 2010 were used for data processing. Quantification of naturally occurring NAPE species was performed by means of a one‐point calibration using the nonendogenous internal standard PE 18:1/18:1‐N‐19:0 (see also Section 2.3).

### 2.8 Method validation

Five rat brains were homogenized with a BioPulverizer (BioSpec Products, Bartlesville, OK, USA) under liquid nitrogen and the homogenate partitioned into aliquots of approximately 100 mg. For evaluation of linearity, the aliquots were spiked with nonendogenous heptadecanoyl NAPEs at nine concentration levels ranging from 10 to 2300 pmol/g. Accuracy was determined by three additional concentration points corresponding to 50, 400, and 1400 pmol/g. All concentration points were fully processed in triplicate as described in Sections 2.3–2.7.

## 3 Results and discussion

### 3.1 Nomenclature

Neither the Lipid Maps nomenclature 37, 38 nor widely accepted existing MS specific shorthand lipidomic nomenclature systems 39 currently include NAPEs. We therefore propose the following nomenclature based on 39, defining NAPE species as derived from *N*‐acylation of the corresponding PE molecules: e.g. PE 36:1‐N‐16:0 when the identities of the O‐acyl groups are not known, or PE 18:0/18:1‐N‐16:0 when the positions of all fatty acyl chains are known.

### 3.2 Synthesis of *N*‐acylphosphatidylethanolamine standards

Because commercial availability of suitable NAPE standards for sample preparation, chromatography, and detection is limited, we synthesized our own standards by means of an esterification reaction using palmitoyl, heptadecanoyl, steaoryl, and arachidonoyl chloride, thus generating the corresponding NAPEs from a natural PE mixture. While the palmitoyl, steaoryl, and arachidonoyl NAPEs were used for chromatographic and mass spectrometric method development, the heptadecanoyl NAPEs, due to their absence in biological tissues, were used for method validation in terms of linearity, accuracy, and efficiency of the sample preparation method.

### 3.3 Development of sample preparation method

Analysis of low‐abundance compounds such as NAPEs is often impaired by other biomolecules present in much greater abundance. Aside from possible ion suppression effects, the extract must then be highly diluted to avoid precipitation of sample components, thus effectively reducing the concentration of the analytes of interest. A total lipid extract from 100 mg brain, which is an extremely lipid‐rich tissue, would have to be diluted in a volume of 10 mL or more for routine lipidomic analysis. While other analytical methods applied to NAPE skip extensive sample preparation methods 6, 7, 30, 32, 34, the presented sample cleanup by SPE eliminates many highly abundant lipid classes such as triacylglycerols that consequently enables reconstitution of the NAPE‐containing fraction in a much smaller volume (200 μL). Preliminary testing during method development showed no analyte losses caused by SPE sample preparation.

### 3.4 *N*‐Acylphosphatidylethanolamine detection and quantification

**Figure 1:**
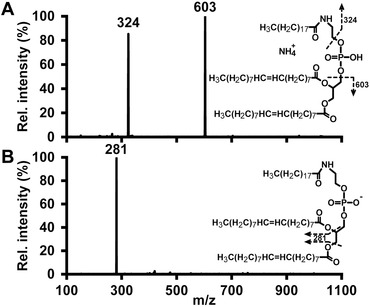
Tandem mass spectra of internal standard PE 18:1/18:1‐N‐19:0 in (A) positive ion mode and (B) negative ion mode at collision energies of 20 eV.

Collision‐induced dissociation of the deprotonated molecule in negative ESI produces ions deriving from the *sn*‐1 and/or *sn*‐2 fatty acyl, but not the *N*‐linked fatty acyl chain (Fig. 1B). While Hansen et al. 40 have previously shown multiple fragment ions derived from collisional activation of the deprotonated molecule, we attribute this observed difference in the fragment spectra to the different types of instruments used (i.e. ion trap and triple quadrupole mass spectrometers), which is consistent with the observations by Larsen et al on PE fragmentation 41. Fragmentation of the ammonium adduct or protonated molecule in positive ESI generates two product ions specific for the *N*‐linked fatty acyl chain, which correspond to cleavage of the C–O bonds on either side of the phosphate group (Fig. 1A), similarly to *N*‐acylphosphatidylserine 42. While other approaches to NAPE determination rely on either full scan, SIM, or a single SRM transition per analyte 6, 7, 25, 26, 27, 28, 29, 30, 31, our method uses these two *N*‐acyl specific transitions for each analyte, leading to a more selective identification and minimizing the risk of false positive results, as shown in Supporting Information Fig. S1.

Another important criterion for correct identification of NAPE species is retention time. With the RP chromatography used, retention increases with the total number of carbon atoms, while double bonds lead to a decrease in retention, as described by the equivalent carbon number model for lipids 43 and demonstrated in Supporting Information Fig. S2. Construction of homologous series, using the internal standard or known retention times of synthesized NAPEs, adds identification certainty in the chromatographic dimension.

**Figure 2:**
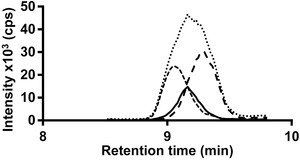
Extracted ion chromatograms of isobaric NAPE structural isomers. Selective detection is enabled by N‐acyl chain specific selected reaction monitoring of PE 38:2‐N‐16:0 (small dashes), PE 36:2‐N‐18:0 (continuous line), and PE 36:1‐N‐18:1 (large dashes). Full scan approaches, even with high mass resolution, would only detect the sum of all structural isomers (dots).

Because NAPEs contain three fatty acyl residues, many possible structural isomers arise from species with different fatty acyl chains. PE 38:2‐N‐16:0, PE 36:2‐N‐18:0, and PE 36:1‐N‐18:1 are isobaric structural isomers with the same elemental composition and therefore cannot be mass separated even by high‐resolution MS. Figure 2 shows the chromatograms of the three aforementioned NAPE species. Although they are partially separated by chromatography, the chromatographic resolution is not sufficient for individual quantification when using only the pseudo‐molecular ion, regardless of mass resolution. SRM in negative ion mode, using the neutral loss or carboxylate ion of the *sn*‐2 fatty acyl chain, also does not resolve ambiguity, since the identities of the *N*‐acyl (and the *sn*‐1) chain remains unknown 27, 28. This shortcoming can be circumvented by *N*‐acyl specific SRM transitions in positive ion mode, enabling identification of the *N*‐acyl chain and the phosphatidylethanolamine moiety.

Preliminary experiments were conducted on pooled rat brain to reduce the possible number of SRM transitions, yielding 73 endogenous NAPE species as candidates for respective SRM construction. The final mass spectrometric method additionally contained SRM transitions for five nonendogenous N‐17:0 NAPEs added for purposes of method validation, and for the internal standard PE 18:1/18:1‐N‐19:0.

**Figure 3:**
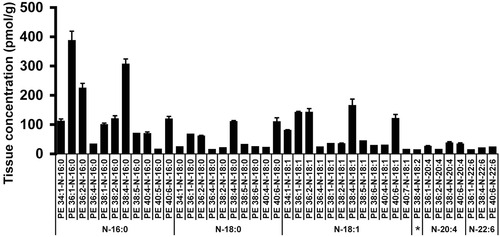
NAPE species quantified in rat brain. Values are means (*n* = 3), error bars represent single SD, asterisk annotates N‐18:2 species.

From 100 mg of rat brain, 41 NAPE species, with a total of six *N*‐acyl fatty acyl chains were quantified (Fig. 3 and Supporting Information Table S1). The concentration of NAPE species found in the investigated tissue amounting to 3 nmol/g is comparable to other published values for rat brain 1, 5, 22, 44, 45, rendering NAPE a low‐abundance lipid such as lysophosphatidic acid or sphingosine‐1‐phosphate 46, 47.

### 3.5 Method validation

Evaluation of linearity was performed by addition of heptadecanoyl‐NAPEs to rat brain samples at concentration levels ranging from 10 to 2300 pmol/g. While all compounds investigated show satisfactory linearity over the whole concentration range (*R* ^2^>0.99), they differ considerably in sensitivity, as indicated by slope values from 0.5 to 1.1 (Table 2). S/Ns at the lowest point of the calibration curve exceed 10:1, a value generally referred to in literature, thus defining the LOQ at 10 pmol/g. Accuracy was evaluated by fitting the response ratios of three additional concentration points (low, 50 pmol/g; medium, 400 pmol/g; high, 1400 pmol/g) into the linear equation derived from the other concentration points. Accuracy was 16.6% at the low concentration level, 7.5% at the medium concentration level, and 14.2% at the high concentration level. Precision was evaluated by calculating the RSDs of tissue concentrations of 41 biological NAPEs from 15 separately processed liver replicates. Generally, values of imprecision are higher at lower concentrations, but for species at concentrations above 25 pmol/g (corresponding to 91% of the total NAPE amount detected), RSD was always under 20%.

**Table 2:** Linearity data (unweighted) for heptadecanoyl‐NAPEs. All values are derived from three separately processed samples for each of the nine concentration points ranging from 10 to 2300 pmol/g

Analyte	Slope	Intercept	*R* ^2^
PE 34:1‐N‐17:0	0.7351	2.2269	0.9919
PE 34:2‐N‐17:0	0.5178	0.7397	0.9901
PE 36:1‐N‐17:0	0.6628	4.1666	0.9944
PE 36:2‐N‐17:0	0.8528	3.6243	0.9942
PE 38:4‐N‐17:0	1.1342	15.414	0.9940

The biggest potential source of error in the whole analytical workflow is sample preparation (both LLE and SPE). This is corroborated by 27% RSD of the internal standard over 39 separately processed samples, underlining its necessity and importance in compensating for variable yields during sample preparation and data acquisition. Due to the extremely limited commercial availability of nonendogenous NAPE compounds and analyte‐free matrices, the use of external calibration curves is not possible, leaving only a label‐free nonendogenous one‐point calibration using an internal standard as a suitable method for quantification.

## 4 Concluding remarks

This method is a substantial improvement of so far existing methodology for determination of NAPE species. This is based on the fact that it relies on the determination of intact NAPE species instead of enzymatically or chemically pretreated fragments thereof, and that LC–MS/MS analysis includes rather two than one compound specific SRM transitions for unambiguous identification of both the *N*‐acyl and the PE moiety. Additionally, sample pretreatment by LLE and SPE minimizes potential interfering matrix compounds, and consequently increases identification certainty. While it is not intended to be a fast high throughput screening method, it is suitable for detection and quantification of physiologically highly relevant signaling precursors in minute amounts. Hence, the scope of this method is lipid rich biological matrices containing many potential interferences, such as rat brain, where 41 intact NAPE species totaling a concentration of 3 nmol/g could be quantified.

*All the authors have declared no conflict of interest*.

## Supporting information

Supporting Material
