# Supplementary material for: Quantitative analysis of N‐acylphosphatidylethanolamine molecular species in rat brain using solid‐phase extraction combined with reversed‐phase chromatography and tandem mass spectrometry
Source: J Sep Sci. 2016 Jun 7;39(13):2474–80. doi: 10.1002/jssc.201600172 (PMC4949747; doi:10.1002/jssc.201600172)
Supplement: Supplementary file 1 — Supporting Material [file JSSC-39-2474-s001.docx]

Supplementary Figure 1: Extracted ion chromatograms (XIC) of PE 36:2‑N‑18:1, y‑axis zoomed to intensity of the correct peak. The dotted line shows the XIC of the N‑acyl fragment while the dashed line shows the XIC of the N‑acyl neutral loss. The correct peak at 6.8 min shows both XICs at the same retention time and comparable intensity. Note that the chromatographic gradient was different from the final method, resulting in a slight shift in retention times.





Supplementary Figure 2: Extracted ion chromatograms of protonated NAPE molecular ions (Panel A: PE 36:1‑N‑16:0, Panel B: PE 36:1‑N‑17:0, Panel C: PE 36:1‑N‑18:0, Panel D: PE 36:2‑N‑18:0). Note the increase of retention time with increasing number of carbon atoms (Panels A, B, C) and the decrease of retention time with increasing number of double bonds (Panels C, D).

Supplementary Table 1: Concentrations of NAPE species detected in rat brain (means and standard deviation from three separately processed samples) and SRM parameters for NAPE detection. Q1 and Q3 resolution was 2 m/z, collision gas argon with a pressure of 1.5 mtorr and retention time window 3 min around the expected retention time.

| Compound name | Tissue concentration (pmol/g) | SD (pmol/g) | Retention time (min) | Q1 mass  (Da) | Q3 mass  (Da) | Collision energy (eV) | Dwell time (ms) |
| --- | --- | --- | --- | --- | --- | --- | --- |
| PE 36:2-N-19:0 (internal standard) | – | – | 9.8 | 1041.80 | 603.48 | -19 | 33.3 |
|  |  |  |  | 1041.80 | 324.33 | -29 | 33.3 |
| PE 34:0-N-16:0 | <LOQ | – | 9.1 | 975.81 | 579.53 | -19 | 24.4 |
|  |  |  |  | 975.81 | 282.28 | -29 | 24.4 |
| PE 34:1-N-16:0 | 107.65 | 11.6 | 7.8 | 973.79 | 577.52 | -19 | 22.9 |
|  |  |  |  | 973.79 | 282.28 | -29 | 22.9 |
| PE 36:0-N-16:0 | <LOQ | – | 10.1 | 1003.84 | 607.57 | -19 | 33.3 |
|  |  |  |  | 1003.84 | 282.28 | -29 | 33.3 |
| PE 36:1-N-16:0 | 383.36 | 35.9 | 9.0 | 1001.83 | 605.55 | -19 | 24.4 |
|  |  |  |  | 1001.83 | 282.28 | -29 | 24.4 |
| PE 36:2-N-16:0 | 220.73 | 20.2 | 7.9 | 999.81 | 603.53 | -19 | 22.9 |
|  |  |  |  | 999.81 | 282.28 | -29 | 22.9 |
| PE 36:4-N-16:0 | 29.99 | 2.5 | 6.6 | 995.78 | 599.50 | -19 | 23.7 |
|  |  |  |  | 995.78 | 282.28 | -29 | 23.7 |
| PE 38:0-N-16:0 | <LOQ | – | 11.2 | 1031.87 | 635.60 | -19 | 56.4 |
|  |  |  |  | 1031.87 | 282.28 | -29 | 56.4 |
| PE 38:1-N-16:0 | 96.05 | 9.1 | 10.1 | 1029.86 | 633.58 | -19 | 33.3 |
|  |  |  |  | 1029.86 | 282.28 | -29 | 33.3 |
| PE 38:2-N-16:0 | 116.02 | 14.5 | 9.0 | 1027.84 | 631.57 | -19 | 24.4 |
|  |  |  |  | 1027.84 | 282.28 | -29 | 24.4 |
| PE 38:4-N-16:0 | 302.64 | 21.8 | 7.7 | 1023.81 | 627.53 | -19 | 22.9 |
|  |  |  |  | 1023.81 | 282.28 | -29 | 22.9 |
| PE 38:5-N-16:0 | 66.31 | 1.1 | 6.7 | 1021.79 | 625.52 | -19 | 23.7 |
|  |  |  |  | 1021.79 | 282.28 | -29 | 23.7 |
| PE 40:4-N-16:0 | 65.51 | 9.3 | 8.5 | 1051.84 | 655.57 | -19 | 24.4 |
|  |  |  |  | 1051.84 | 282.28 | -29 | 24.4 |
| PE 40:5-N-16:0 | 12.26 | 1.4 | 8.0 | 1049.83 | 653.55 | -19 | 22.9 |
|  |  |  |  | 1049.83 | 282.28 | -29 | 22.9 |
| PE 40:6-N-16:0 | 115.24 | 12.7 | 7.2 | 1047.81 | 651.53 | -19 | 22.9 |
|  |  |  |  | 1047.81 | 282.28 | -29 | 22.9 |
| PE 34:1-N-17:0 | – | – | 8.4 | 987.81 | 577.52 | -19 | 23.7 |
|  |  |  |  | 987.81 | 296.29 | -29 | 23.7 |
| PE 34:2-N-17:0 | – | – | 7.3 | 985.79 | 575.50 | -19 | 22.9 |
|  |  |  |  | 985.79 | 296.29 | -29 | 22.9 |
| PE 36:1-N-17:0 | – | – | 9.6 | 1015.84 | 605.55 | -19 | 31.9 |
|  |  |  |  | 1015.84 | 296.29 | -29 | 31.9 |
| PE 36:2-N-17:0 | – | – | 8.6 | 1013.83 | 603.53 | -19 | 24.4 |
|  |  |  |  | 1013.83 | 296.29 | -29 | 24.4 |
| PE 38:4-N-17:0 | – | – | 8.3 | 1037.83 | 627.53 | -19 | 22.9 |
|  |  |  |  | 1037.83 | 296.29 | -29 | 22.9 |
| PE 34:0-N-18:0 | <LOQ | – | 10.0 | 1003.84 | 579.53 | -19 | 33.3 |
|  |  |  |  | 1003.84 | 310.31 | -29 | 33.3 |
| PE 34:1-N-18:0 | 20.61 | 1.3 | 9.0 | 1001.83 | 577.52 | -19 | 24.4 |
|  |  |  |  | 1001.83 | 310.31 | -29 | 24.4 |
| PE 36:0-N-18:0 | <LOQ | – | 11.2 | 1031.87 | 607.57 | -19 | 56.4 |
|  |  |  |  | 1031.87 | 310.31 | -29 | 56.4 |
| PE 36:1-N-18:0 | 63.64 | 3.3 | 10.2 | 1029.86 | 605.55 | -19 | 33.3 |
|  |  |  |  | 1029.86 | 310.31 | -29 | 33.3 |
| PE 36:2-N-18:0 | 56.87 | 6.4 | 9.1 | 1027.84 | 603.53 | -19 | 24.4 |
|  |  |  |  | 1027.84 | 310.31 | -29 | 24.4 |
| PE 36:4-N-18:0 | 11.26 | 1.8 | 7.7 | 1023.81 | 599.50 | -19 | 22.9 |
|  |  |  |  | 1023.81 | 310.31 | -29 | 22.9 |
| PE 38:1-N-18:0 | <LOQ | – | 11.3 | 1057.89 | 633.58 | -19 | 66.7 |
|  |  |  |  | 1057.89 | 310.31 | -29 | 66.7 |
| PE 38:2-N-18:0 | 17.27 | 3.0 | 10.2 | 1055.87 | 631.57 | -19 | 33.3 |
|  |  |  |  | 1055.87 | 310.31 | -29 | 33.3 |
| PE 38:4-N-18:0 | 106.13 | 7.7 | 8.9 | 1051.84 | 627.53 | -19 | 24.4 |
|  |  |  |  | 1051.84 | 310.31 | -29 | 24.4 |
| PE 38:5-N-18:0 | 28.29 | 3.0 | 7.8 | 1049.83 | 625.52 | -19 | 22.9 |
|  |  |  |  | 1049.83 | 310.31 | -29 | 22.9 |
| PE 38:6-N-18:0 | 20.72 | 2.4 | 7.2 | 1047.81 | 623.50 | -19 | 22.9 |
|  |  |  |  | 1047.81 | 310.31 | -29 | 22.9 |
| PE 40:4-N-18:0 | 18.23 | 1.4 | 9.7 | 1079.87 | 655.57 | -19 | 31.9 |
|  |  |  |  | 1079.87 | 310.31 | -29 | 31.9 |
| PE 40:5-N-18:0 | <LOQ | – | 9.3 | 1077.86 | 653.55 | -19 | 25.3 |
|  |  |  |  | 1077.86 | 310.31 | -29 | 25.3 |
| PE 40:6-N-18:0 | 105.68 | 17.8 | 8.3 | 1075.84 | 651.53 | -19 | 22.9 |
|  |  |  |  | 1075.84 | 310.31 | -29 | 22.9 |
| PE 34:1-N-18:1 | 76.29 | 6.3 | 8.0 | 999.81 | 577.52 | -19 | 22.9 |
|  |  |  |  | 999.81 | 308.29 | -29 | 22.9 |
| PE 36:0-N-18:1 | <LOQ | – | 10.2 | 1029.86 | 607.57 | -19 | 33.3 |
|  |  |  |  | 1029.86 | 308.29 | -29 | 33.3 |
| PE 36:1-N-18:1 | 137.73 | 7.4 | 9.2 | 1027.84 | 605.55 | -19 | 24.4 |
|  |  |  |  | 1027.84 | 308.29 | -29 | 24.4 |
| PE 36:2-N-18:1 | 138.32 | 16.4 | 8.1 | 1025.83 | 603.53 | -19 | 22.9 |
|  |  |  |  | 1025.83 | 308.29 | -29 | 22.9 |
| PE 36:4-N-18:1 | 20.07 | 1.6 | 6.7 | 1021.79 | 599.50 | -19 | 22.9 |
|  |  |  |  | 1021.79 | 308.29 | -29 | 22.9 |
| PE 38:1-N-18:1 | 31.63 | 3.3 | 10.3 | 1055.87 | 633.58 | -19 | 33.3 |
|  |  |  |  | 1055.87 | 308.29 | -29 | 33.3 |
| PE 38:2-N-18:1 | 30.69 | 6.2 | 9.2 | 1053.86 | 631.57 | -19 | 24.4 |
|  |  |  |  | 1053.86 | 308.29 | -29 | 24.4 |
| PE 38:3-N-18:1 | <LOQ | – | 8.8 | 1051.84 | 629.55 | -19 | 24.4 |
|  |  |  |  | 1051.84 | 308.29 | -29 | 24.4 |
| PE 38:4-N-18:1 | 161.29 | 26.0 | 7.9 | 1049.83 | 627.53 | -19 | 22.9 |
|  |  |  |  | 1049.83 | 308.29 | -29 | 22.9 |
| PE 38:5-N-18:1 | 40.51 | 2.2 | 6.9 | 1047.81 | 625.52 | -19 | 22.9 |
|  |  |  |  | 1047.81 | 308.29 | -29 | 22.9 |
| PE 38:6-N-18:1 | 25.01 | 1.1 | 6.3 | 1045.79 | 623.50 | -19 | 25.3 |
|  |  |  |  | 1045.79 | 308.29 | -29 | 25.3 |
| PE 40:2-N-18:1 | <LOQ | – | 10.4 | 1081.89 | 659.60 | -19 | 33.3 |
|  |  |  |  | 1081.89 | 308.29 | -29 | 33.3 |
| PE 40:4-N-18:1 | 26.09 | 2.0 | 8.7 | 1077.86 | 655.57 | -19 | 24.4 |
|  |  |  |  | 1077.86 | 308.29 | -29 | 24.4 |
| PE 40:5-N-18:1 | <LOQ | – | 8.3 | 1075.84 | 653.55 | -19 | 22.9 |
|  |  |  |  | 1075.84 | 308.29 | -29 | 22.9 |
| PE 40:6-N-18:1 | 116.96 | 17.7 | 7.4 | 1073.83 | 651.53 | -19 | 22.9 |
|  |  |  |  | 1073.83 | 308.29 | -29 | 22.9 |
| PE 40:7-N-18:1 | 11.34 | 1.0 | 6.4 | 1071.81 | 649.52 | -19 | 24.4 |
|  |  |  |  | 1071.81 | 308.29 | -29 | 24.4 |
| PE 34:1-N-18:2 | <LOQ | – | 7.1 | 997.79 | 577.52 | -19 | 22.9 |
|  |  |  |  | 997.79 | 306.28 | -29 | 22.9 |
| PE 36:1-N-18:2 | <LOQ | – | 8.2 | 1025.83 | 605.55 | -19 | 22.9 |
|  |  |  |  | 1025.83 | 306.28 | -29 | 22.9 |
| PE 36:2-N-18:2 | <LOQ | – | 7.2 | 1023.81 | 603.53 | -19 | 22.9 |
|  |  |  |  | 1023.81 | 306.28 | -29 | 22.9 |
| PE 38:4-N-18:2 | 10.11 | 1.7 | 7.0 | 1047.81 | 627.53 | -19 | 22.9 |
|  |  |  |  | 1047.81 | 306.28 | -29 | 22.9 |
| PE 38:6-N-18:2 | <LOQ | – | 5.4 | 1043.78 | 623.50 | -19 | 40.7 |
|  |  |  |  | 1043.78 | 306.28 | -29 | 40.7 |
| PE 40:6-N-18:2 | <LOQ | – | 6.5 | 1071.81 | 651.53 | -19 | 23.7 |
|  |  |  |  | 1071.81 | 306.28 | -29 | 23.7 |
| PE 34:1-N-20:4 | <LOQ | – | 6.9 | 1021.79 | 577.52 | -19 | 22.9 |
|  |  |  |  | 1021.79 | 330.28 | -29 | 22.9 |
| PE 36:0-N-20:4 | <LOQ | – | 9.3 | 1051.84 | 607.57 | -19 | 24.4 |
|  |  |  |  | 1051.84 | 330.28 | -29 | 24.4 |
| PE 36:1-N-20:4 | 22.02 | 6.3 | 8.1 | 1049.83 | 605.55 | -19 | 22.9 |
|  |  |  |  | 1049.83 | 330.28 | -29 | 22.9 |
| PE 36:2-N-20:4 | 11.47 | 2.1 | 7.1 | 1047.81 | 603.53 | -19 | 22.9 |
|  |  |  |  | 1047.81 | 330.28 | -29 | 22.9 |
| PE 36:3-N-20:4 | <LOQ | – | 6.1 | 1045.79 | 601.52 | -19 | 26.2 |
|  |  |  |  | 1045.79 | 330.28 | -29 | 26.2 |
| PE 38:1-N-20:4 | <LOQ | – | 9.2 | 1077.86 | 633.58 | -19 | 24.4 |
|  |  |  |  | 1077.86 | 330.28 | -29 | 24.4 |
| PE 38:2-N-20:4 | <LOQ | – | 8.1 | 1075.84 | 631.57 | -19 | 22.9 |
|  |  |  |  | 1075.84 | 330.28 | -29 | 22.9 |
| PE 38:3-N-20:4 | <LOQ | – | 7.4 | 1073.83 | 629.55 | -19 | 22.9 |
|  |  |  |  | 1073.83 | 330.28 | -29 | 22.9 |
| PE 38:4-N-20:4 | 33.24 | 8.5 | 6.9 | 1071.81 | 627.53 | -19 | 22.9 |
|  |  |  |  | 1071.81 | 330.28 | -29 | 22.9 |
| PE 40:4-N-20:4 | <LOQ | – | 7.6 | 1099.84 | 655.57 | -19 | 22.9 |
|  |  |  |  | 1099.84 | 330.28 | -29 | 22.9 |
| PE 40:5-N-20:4 | <LOQ | – | 7.2 | 1097.83 | 653.55 | -19 | 22.9 |
|  |  |  |  | 1097.83 | 330.28 | -29 | 22.9 |
| PE 40:6-N-20:4 | 29.85 | 8.1 | 6.3 | 1095.81 | 651.53 | -19 | 24.4 |
|  |  |  |  | 1095.81 | 330.28 | -29 | 24.4 |
| PE 34:0-N-22:6 | <LOQ | – | 7.5 | 1047.81 | 579.53 | -19 | 22.9 |
|  |  |  |  | 1047.81 | 354.28 | -29 | 22.9 |
| PE 34:1-N-22:6 | <LOQ | – | 6.5 | 1045.79 | 577.52 | -19 | 24.4 |
|  |  |  |  | 1045.79 | 354.28 | -29 | 24.4 |
| PE 36:1-N-22:6 | 10.36 | 2.9 | 7.7 | 1073.83 | 605.55 | -19 | 22.9 |
|  |  |  |  | 1073.83 | 354.28 | -29 | 22.9 |
| PE 36:2-N-22:6 | <LOQ | – | 6.7 | 1071.81 | 603.53 | -19 | 23.7 |
|  |  |  |  | 1071.81 | 354.28 | -29 | 23.7 |
| PE 36:4-N-22:6 | <LOQ | – | 5.4 | 1067.78 | 599.50 | -19 | 40.7 |
|  |  |  |  | 1067.78 | 354.28 | -29 | 40.7 |
| PE 38:4-N-22:6 | 16.78 | 2.2 | 6.5 | 1095.81 | 627.53 | -19 | 23.7 |
|  |  |  |  | 1095.81 | 354.28 | -29 | 23.7 |
| PE 38:5-N-22:6 | <LOQ | – | 5.5 | 1093.79 | 625.52 | -19 | 36.7 |
|  |  |  |  | 1093.79 | 354.28 | -29 | 36.7 |
| PE 38:6-N-22:6 | <LOQ | – | 5.0 | 1091.78 | 623.50 | -19 | 73.3 |
|  |  |  |  | 1091.78 | 354.28 | -29 | 73.3 |
| PE 40:5-N-22:6 | <LOQ | – | 6.9 | 1121.83 | 653.55 | -19 | 22.9 |
|  |  |  |  | 1121.83 | 354.28 | -29 | 22.9 |
| PE 40:6-N-22:6 | 19.64 | 1.2 | 6.0 | 1119.81 | 651.53 | -19 | 27.2 |
|  |  |  |  | 1119.81 | 354.28 | -29 | 27.2 |
| PE 40:7-N-22:6 | <LOQ | – | 5.1 | 1117.79 | 649.52 | -19 | 61.1 |
|  |  |  |  | 1117.79 | 354.28 | -29 | 61.1 |
